# Aneurismal Tumor, Situated between the Brain and Sella Tursica

**Published:** 1827-12

**Authors:** Thomas William Chevalier

**Affiliations:** Consulting Surgeon to the Royal Union Association, and Surgeon to the Westminster General Dispensary.


### PATHOLOGY OF THE BRAIN.

Case I. *Aneurismal Tumor, situated between the Brain and Sella Turcica.* By Thomas William Chevalier, Consulting Surgeon to the Royal Union Association, and Surgeon to the Westminster General Dispensary.

S. B. ætat. thirty-nine, a woman of masculine form and robust constitution, applied to me on the 8th of October, when I obtained the following history of her case from herself and her mother :— She had suffered from a severe burn at the age of fourteen, and had ever since complained of an unusual sensation, which she described as "a shaking movement in her inside." She had been more or less subject to headache from her infancy, and finally ceased to menstruate seven years since, without experiencing any inconvenience.

She had always been fond of reading, and indulged much in that amusement. Her circumstances obliged her to lead an extremely temperate and laborious life; to which, however, she was cheerfully inclined.

About the month of May last, she first began to be afflicted with a strange sort of headache, very different from that to which she was previously accustomed, and a sensation of burning heat in the stomach, feeling (to use her own expression,) "as if her inside were on fire," and she frequently complained of the excessive heat of her mouth.

These headaches, which were very severe, usually occurred more than once a week, and lasted, if at night, during two or three hours, but, in the daytime, usually for half an hour.

They were always preceded by flushing of the countenance, and an increased sensation of heat, particularly in the region of the stomach, without any shivering or perspirations. They invariably commenced at the back of the head, the pain shooting through to the right eye, and generally producing œdema of the palpebræ. They were always relieved by the application of cold to the head, and they would at any time recur if she attempted to sit by the fire, or even to expose herself to the heat of the sun.

Her mother informed me that she often seemed to lose her recollection and consciousness for the space of a minute or two during these attacks, and that her health had appeared to decline from last spring, at which time they commenced.

About six weeks since, she remarked that she felt the same kind of "shaking or beating" in her head which until now she had experienced only in her stomach. From this time her head was never entirely free from pain, and her sight became slightly impaired, so that she could not see to thread her needle; and about the same period, while walking on one occasion in Oxford-street, she found herself falling forward to the ground, and would have dropped had she not saved herself by taking hold of the railings. She presently recovered, and returned home, the distance of a furlong, without assistance.

Three weeks before she died, she fell upon her face in the street, and remained insensible for a few minutes; after which she was conducted home, with her usual headache.

On Monday October 8th, she consented, for the first time, to receive medical advice, and accompanied her mother to my house, when I obtained the above account. I found that she retained the senses of hearing and smelling unimpaired, as well as her appetite; her sense of taste had been deranged for months past, but this proceeded, as she thought, from the heat of her mouth. Her countenance was now strongly marked with despondency, and she exhibited an air of stupid indifference, which would have induced me to believe her intoxicated or insane; but, upon inquiry, I found her intellect perfectly sound, and her habits of life most abstemious.

Her pulse ninety-six, labouring, but not hard or full; tongue furred; bowels habitually costive; countenance florid, but as if chilled by cold. I prescribed a bluepill every night, and a compound colocynth pill every morning, and ordered her to be cupped on the back of the neck to sixteen ounces.

On the following morning she was much relieved, and enlivened in her general appearance.

On Tuesday night, however, the pain in the head recurred with increased violence, accompanied by sickness, and continued nearly all night.

In the morning I found her cheerful, and rather improved than otherwise in every respect. To take one ounce of Epsom salts tomorrow morning, and to continue the pills.

October 19th.—She went to bed last night tolerably well. At half-past one o'clock, however, she got out of bed, and awaked her mother, who inquired what she was doing? to which question she replied, that she was opening the shutters to admit the light, because the room was so dark; whereas there was a candle burning in the room, and no shutters whatever to the window. Her mother arose in time to prevent her falling to the ground, and replaced her in bed.

From this time she became comatose, and I was sent for in the forenoon. I found her perfectly devoid of intellect, and almost entirely deprived of sensation and voluntary motion; with a small, rapid, and labouring pulse; the feet cold; the pupil small and fixed; the countenance not in the least distorted; the breathing rather laborious, but not otherwise unnatural. She shrunk from the examination of the pupil, but evinced no other sign of sensation.

**To be cupped to twelve ounces immediately, and a large blister applied to the back of the neck. Spirit lotion to the head; and an enema of compound Extract of Colocynth and gruel as soon as possible.**

At noon she appeared a little revived from the cupping, and uttered a word or two upon being disturbed: her pulse had, however, increased in frequency, with scarcely any augmentation of its strength; and she died at one o'clock on the following morning, without having manifested any return of consciousness, or exhibited any other symptoms than those of pure coma.

*Dissection.*—There were extensive cicatrices upon the arms, neck, and chest. One side of the thorax was considerably depressed by a distortion of many years' standing, but the lungs were free from any unnatural adhesion, and every part of the trunk perfectly healthy.

On opening the head, the vessels of the meninges appeared rather turgid, and an extravasation of recent blood was found beneath the left parietal bone, between the arachnoid membrane and the pia mater, but its quantity did not exceed a table-spoonful, and was diffused over a circular space of three inches diameter. The substance of the brain was unusually firm and healthy. The ventricles contained no more than about one fluid drachm of clear serum.

Upon raising the encephalon from the base of the skull, there was discovered a tumor of the size of a large walnut, attached to the pia mater of the base of the brain, and altogether inseparable without violence, from the sella turcica, so that an attempt was made to remove the adjacent bone, together with the brain, but without success, in consequence of the restraint which the presence of the friends imposed. The soft parts being at length separated, the base of the tumor appeared to have filled the sella turcica, which was left clean by its removal, and its chief bulk had been situated so as to separate the corpora albicantia and the optic nerves more than an inch and a quarter from the posterior clinoid processes. The tumor itself was nearly spherical, inclosed in a fine transparent membrane, excepting where it was cut away from the bone, and of so firm a texture that I was induced to believe it solid throughout. Upon cutting into it, however, after it had been macerated in alcohol for four or five days, I found it to consist entirely of coagula of blood, deposited in several distinct layers, which, like those of an aneurism, were paler on one side, viz. towards the bone, and softer as they approached the corpora albicantia, where there was a cavity as large as a filbert, containing recent coagulum.

With the utmost pains, I have been unable to detect any direct communication between the tumor and the adjacent arterial trunks: three or four comparatively minute branches of the left anastomosing artery of the circle of Willis are, however, so closely connected with its membranous envelope, that one of these may probably have furnished its contents. The preparation not having been injected, it is impossible to determine this point so accurately as might be desired. I hope, however, that the account here given may not be altogether useless, as the symptoms described were sufficient to create a suspicion in my mind, from the first, of the true nature of the disease, and enabled me to foretel to a medical friend the result of the dissection with tolerable accuracy.

20, *South Audley-street; November* 12*th*, 1827.

